# Identifying the Elusive Dimerization Product Interfering with Methylsulfonato‐Group Labeling of Cysteines in Proteins

**DOI:** 10.1002/open.202500314

**Published:** 2025-10-07

**Authors:** Leonardo Passerini, René Dekkers, Karthick Babu Sai Sankar Gupta, Mark Overhand, Martina Huber

**Affiliations:** ^1^ Department of Physics Huygens‐Kamerlingh Onnes Laboratory Leiden University Niels Bohrweg 2 2333 CA Leiden The Netherlands; ^2^ Leiden Insititute of Chemistry Leiden University Einsteinweg 55 2333 CC Leiden The Netherlands

**Keywords:** biradical‐side reaction, electron paramagnetic resonance, methanethiosulfonate, nitroxide spin label, spin‐label reaction

## Abstract

Many biomolecular studies start with labeling a protein with a fluorescent label, spin label, or chemical label. The methanethiosulfonate (mts)‐linking group suffers from a hitherto not‐understood side reaction that leads to label‐dimerization instead of the desired linking of the label to the cysteine of the protein. Using electron paramagnetic resonance and mass spectrometry, the side reaction is studied for the MTSL ((1‐oxyl‐2,2,5,5‐tetramethyl‐Δ‐3‐pyrroline‐3‐methyl) methanethiosulfonate) and the (1‐oxyl‐2,2,5,5‐tetramethylpyrrolidin‐3‐yl) methyl methanethiosulfonate label. At 0.1 mM MTSL, substantial dimer formation is observed within the first 5 h. The reaction pathway is elucidated and the structure of the disulfide‐linked asymmetric dimer is suggested. The reaction seems not to involve the nitroxide or a radical reaction, suggesting that this reaction could also occur for other mts‐linked functional or labeling groups.

## Graphical abstract

Too much of a good thing: At too high concentrations of methylsulfoxide‐(spin‐)labels in the labelling mixture, instead of labelling the cysteine of the protein, the label dimerizes with itself. What is the structure of the dimer? After four decades of puzzle, here is the answer.

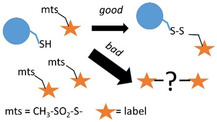

## 1 Introduction

**Figure 1:**
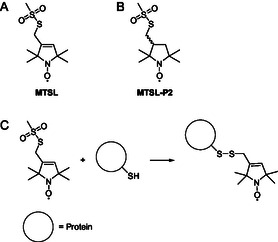
A) Structure of methylsulfoxide spin label (MTSL) investigated, B) MTSL‐P2, and C) labeling‐reaction scheme of the reaction of the label with a cysteine in the protein (C).

The methanethiosulfonate (mts)‐group is an often used functional group to couple an mts‐label to a thiol‐group, often from a cysteine of a protein. The mts‐group, shown in **Figure** **1**, is popular because of its high reactivity and selectivity. It enables to selectively label cysteines of proteins with spin or fluorescent labels. To illustrate the broad range of the use of the mts group in spin‐label research, here some recent examples.^[^ ^1^, ^2^, ^3^, ^4^, ^5^ ^–^ ^6^ ^]^ An overview of other linking groups is given in some recent publications.^[^ ^7^, ^8^ ^–^ ^9^ ^]^ Further, recent developments circumvent cysteine labeling completely to enable labeling in cellular contexts; see for example ref. [10].

Often, labeling is the first step to obtain structural or biochemical information, because it opens up a way to apply a variety of techniques in biochemical research, such as electron paramagnetic resonance (EPR), super‐resolution‐microscopy‐techniques, or Förster‐resonance‐energy‐transfer (FRET) based distance measurements. Spin labeling is universally applied to investigate proteins by EPR or paramagnetic NMR.^[^ ^11^ ^,^ ^12^ ^]^ It enables structure determination by nanometer‐distance constraints by EPR methods^[^ ^13^, ^14^, ^15^ ^–^ ^16^ ^]^ or the elucidation of protein dynamics or protein‐partner interactions, in vitro or in cells.^[^ ^1^ ^,^ ^17^, ^18^, ^19^, ^20^, ^21^ ^–^ ^22^ ^]^

Yet the reaction has a major disadvantage: Under standard conditions, when cysteine groups of proteins are labeled with the well‐known spin label MTSL ((1‐oxyl‐2,2,5,5‐tetramethyl‐Δ‐3‐pyrroline‐3‐methyl) methanethiosulfonate),^[^ ^23^ ^]^ a side reaction occurs that limits the reaction yield. A characteristic EPR signal shows that the side product is a molecule in which two nitroxide radicals are coupled, that is, a nitroxide‐biradical. To suppress this reaction, the MTSL concentrations has to be kept below 200 µM,^[^ ^24^ ^]^ which limits protein concentrations to 20 µM at the typical 10:1 excess of spin label. While for many proteins this may not be considered a severe restriction, in other cases, the additional dilution and concentration steps required may interfere with protein stability or reduce the yield of labeled protein.

In spite of four decades of site‐directed‐mutagenesis spin labeling, pioneered by the Hubbell group,^[^ ^19^ ^,^ ^20^ ^]^ the underlying reaction is not known. Here, we study the formation of the side‐product under typical spin‐labeling conditions for the most‐used variants of the methanethiosulfonate spin label (Figure 1A,B) and determine the structure of the biradical‐side product by a combination of EPR and mass spectrometry, solving a puzzle that was plaguing labeling reactions for the last four decades.

## 2 Results

In a typical spin‐labeling reaction the protein is incubated with a 10‐fold excess of spin label in a suitable aqueous buffer solution using reaction times between 2 and 18 h, either at room temperature or at 4 °C. Here we mirror these conditions by studying the reaction systematically in the absence of the protein.

**Figure 2:**
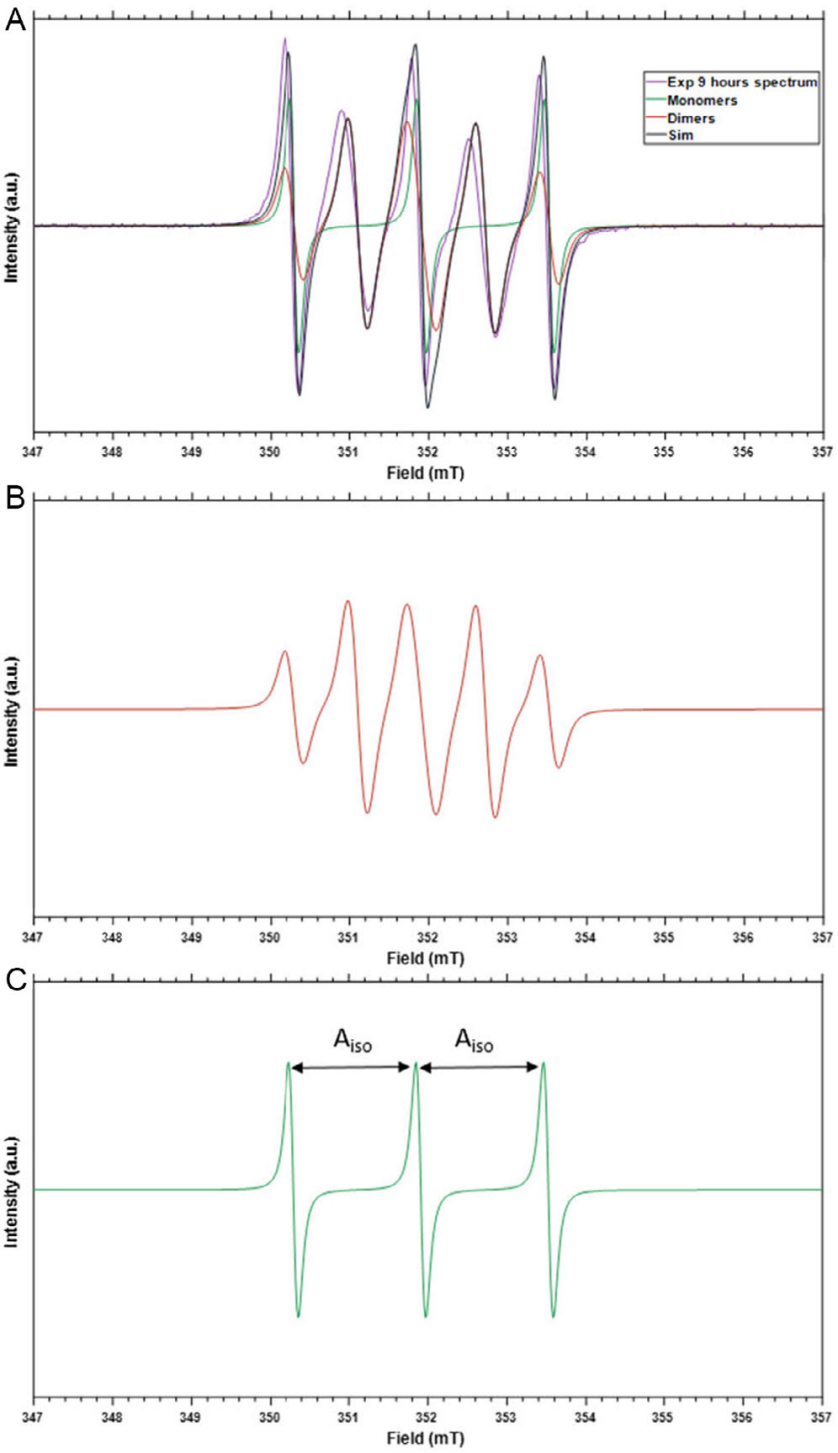
Formation of the MTSL biradical monitored by EPR. A) Spectrum, after 9 h of incubation in aqueous buffer. Purple: experimental spectrum, black: total simulation, orange: biradical component, green: monoradical component. B) Biradical component from (A). C) Monoradical component. EPR spectra recorded as first derivative of absorption.

**Figure** **2** shows the EPR spectrum of (1‐oxyl‐2,2,5,5‐tetramethyl‐Δ‐3‐pyrroline‐3‐methyl) methanethiosulfonate (MTSL) after 9 h of incubation in an aqueous solution (buffer), such as the conditions normally used for protein‐spin labeling. The spectrum observed is the superposition of two components, shown separately in Figure 2B,C. One is the classical three‐line spectrum (Figure 2B) of nitroxide radicals at room temperature. The splitting into three lines arises from hyperfine interaction of the unpaired electron with the ^14^N nucleus of the N—O bond. The second component is a five‐line spectrum (Figure 2C) that originates from two coupled nitroxide radicals,^[^ ^25^ ^]^ which we will refer to in the following as biradical. A five‐line pattern is observed if the exchange interaction (*J*) is significantly larger than the hyperfine interaction *A*_iso_ (*J* > 2 *A*_iso_).^[^ ^25^, ^26^ ^–^ ^27^ ^]^ In **Table** **1**, the relevant spectral parameters are given, which are obtained from spectral simulations (see Experimental Section).

**Table 1:** Parameters used for simulation of mono‐ and biradical EPR spectral components of MTSL and MTSL‐P2. For details, see Experimental Section.

	Monomer		Biradical		
	*g*_iso_	*A*_iso_ [MHz]	*g*_iso_	*A*_iso_ [MHz]	*J* [MHz]
MTSL	2.0059	45.3	2.0059	45.3	600
MTSL‐P2	2.0059	45.3	2.0059	45.3	71

**Figure 3:**
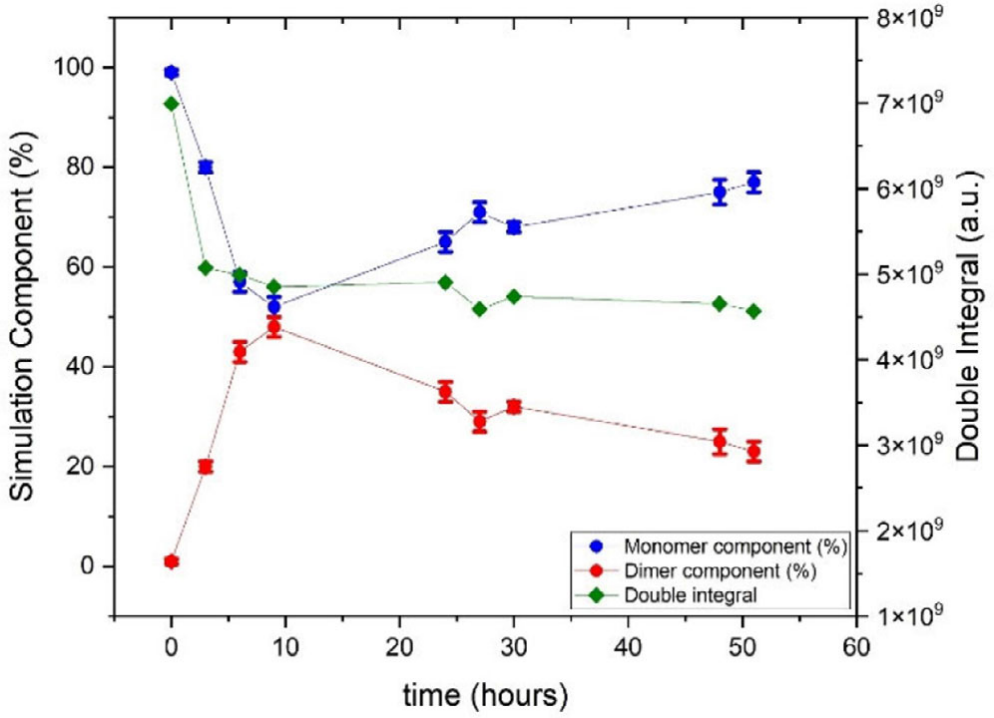
Development of mono‐ and biradical components for MTSL over time. Blue: monoradical contribution, red: biradical contribution, green: total intensity of the EPR spectrum (double integral). For details about error estimation, see Experimental Section.

Spectra were acquired in time intervals over the course of two days. **Figure** **3** shows the relative contribution of the MTSL biradical and the monomer component as they develop over time. The biradical contribution increases over time and a maximum of 55% in the biradical component is observed after 9 h of incubation, followed by a decrease to a value of 23% after 51 h of incubation. The total radical concentration, obtained from the double integration of the EPR spectra, shows an initial decrease in the number of spins in the sample during the first 3 h of incubation, possibly due to decay of the radical. Then, the number of spins in the sample reaches a plateau and remains constant for the entire incubation time (green points in Figure 3).

**Figure 4:**
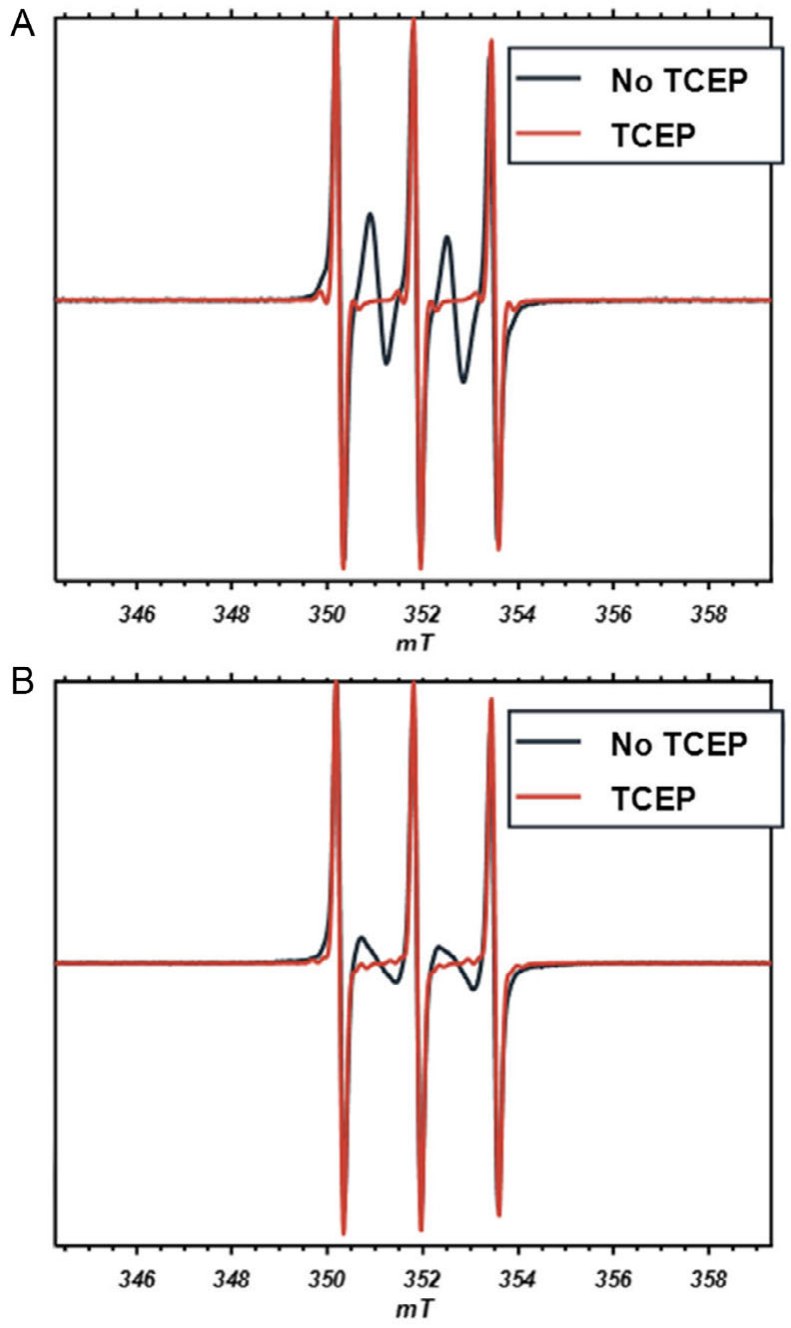
Reductant TCEP (tris(2‐carboxyethyl)phosphine) abolishes biradical component. Effect of TCEP on biradical component of A) MTSL and B) MTSL‐P2. Black: spectrum after 3 h of incubation, orange: spectrum obtained after addition of excess TCEP.

After a fair amount (43%) of biradical had formed in the solution, a mild reductant, specific for disulfide bridges, TCEP (tris(2‐carboxyethyl)phosphine), reaction seen in Figure S6 (Supporting Information), was added to the incubating MTSL solution in a fivefold excess relative to MTSL. This abolishes the biradical component in the EPR spectrum completely (see **Figure** **4**A), suggesting that the biradical contains a disulfide bond.

**Figure 5:**
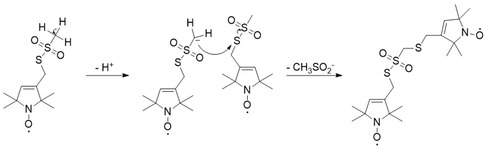
Proposed reaction mechanism for MTSL and structure of the dimer product (right). Note: The additional oxygen‐atom mass of the base peak (Table 2) is attributed to sulfur oxidation at a cysteine.^[^ ^30^ ^]^

To obtain information on the structure of the dimer, we used mass spectrometry (electron‐spray‐ionization mass spectrometry, ESI‐MS). Mass spectrometry of the MTSL sample after 3 h of incubation shows a base peak at 466 m/z. Simulation of the peak gives the element composition of the species: C_19_H_34_O_5_N_2_S_3._ Upon addition of TCEP to the sample, the 466 m/z peak disappears, and a new base peak of 267 m/z appears, corresponding to the oxidized TCEP. The peak of 267 m/z confirms that TCEP reacts with a disulfide bond (reaction scheme **Figure** **5**). Another new peak at 249  m/z is observed after reaction of the dimer with TCEP, possibly a breakdown product of the reaction between the MTSL dimer and TCEP; however, this peak could not be assigned to a specific chemical structure. Mass spectra are shown in Figure S5 (Supporting Information), and in **Table** **2**, the base peaks observed are listed.

**Table 2:** Mass spectrometry results: base peaks observed in the mass spectra of MTSL and MTSL‐P2 after 24 h incubation and after TCEP addition to the sample. Mass spectra are shown in Supporting Information. For details, see Experimental Section.

	During incubation			After TCEP addition	
	Massa)		Sum formula	Mass	
MTSL	466.16210	100%	C_19_H_34_O_5_N_2_S_3_	267.06245b)	100%
MTSL‐P2	470.19340	100%	C_19_H_38_O_5_N_2_S_3_	267.06240b)	100%

a) Contain extra O‐atom because of cysteine oxidation.^[^ ^20^ ^]^

b) Oxidized TCEP.

For the analogous compound, MTSL‐P2, similar results to MTSL were observed. In Figure 4B, the EPR spectrum obtained after 3 h incubation is shown. Also in this case, the spectrum originates from a superposition of the three‐line‐nitroxide spectrum with a biradical spectrum of two coupled nitroxides. For MTSL‐P2, the shape of the biradical spectrum differs from that of MTSL. The spectrum can be simulated with *J* = 71 MHz; see Table 1. This value of *J*, however, does not reproduce accurately the experimental spectrum, as the peaks at 345 and 356 mT in the simulation are not observed in the spectrum (see Figure S1, Supporting Information). The time course of the reaction (Figure S4, Supporting Information) closely parallels that of MTSL. Also in the case of MTSL‐P2, addition of TCEP completelely abolished the biradical component in the EPR spectrum; see Figure 4 B.

Mass spectra of the MTSL‐P2 solution after 3 h of incubation show a base peak at 470 m/z, and as observed for MTSL, addition of TCEP causes the disappearance of the 470 m/z peak and appearance of the peak corresponding to oxidized TCEP, at 267 m/z.

For more details on the simulation of the EPR spectra, such as the pure spectral components, simulation, and interpretation of different values of *J* in the two biradicals MTSL and MTSL‐P2, see Supporting Information.

The similar behavior of MTSL and MTSL‐P2 with respect to dimer formation, the reaction with TCEP, and the base peaks of the mass spectra of the dimers observed indicates that the same chemical dimerization reaction is happening for both labels.

## 3 Discussion

In this study, we investigate an unwanted side reaction that interferes with spin labeling of cysteine‐proteins. The goal of the study was to understand the reaction and determine the product. To do so, we investigated conditions of product formation for two different spin labels, MTSL and MTSL‐P2 (Figure 1A,B), and propose structures for the products. For both compounds, we show that the product of the side reaction is a nitroxide‐dimer, in which the two nitroxide moieties are covalently linked in a reaction that irreversibly converts the methyl‐sulfothionate‐ (mts‐)groups of the MTSL molecule.

Within the first 9 h of incubation, about 55% of the MTSL is converted to biradical. The biradical contribution to the spectra remains constant (MTSL‐P2) or decays after the initial growth period (MTSL). We attribute the decay in the latter case to breakage of the biradical product or to a biradical decay, in which one of the nitroxides of the biradical becomes diamagnetic.

The amount of biradical formed in these experiments is higher than one could expect, considering that in spin‐labeling reactions the concentration of MTSL can be twice as large^[^ ^24^ ^]^ as in the experiments described here, and also often longer reaction times are used. In the experiments presented here, however, the cysteine‐bearing protein (see Figure 1) is absent. We suggest that in standard spin‐labeling reactions, the mts‐group reacts faster with the electron‐rich cysteine SH‐group of the protein than with another mts‐group (see below), explaining that in protein‐labeling dimer formation occurs at significantly higher MTSL concentrations than observed here.

Dimer Structure: The EPR spectrum of the biradical contribution shows that two nitroxide moieties interact, and the magnitude of the exchange coupling (*J*) suggests effective through‐space or through‐bond coupling of the two nitroxide groups.^[^ ^25^ ^]^ The biradical of MTSL‐P2 has a smaller *J* than MTSL, suggesting a weaker nitroxide–nitroxide interaction than is the case of MTSL. As described in the Supporting Information, some spectral features do not agree with the precise *J*‐value used, suggesting that the difference in *J* could also be due to differences in the biradical‐conformational dynamics, as described for other nitroxide biradicals before.^[^ ^25^ ^,^ ^27^ ^]^ Therefore, the difference in the EPR spectra of MTSL and MTSL‐P2 cannot be further interpreted. Generally, the exchange interaction cannot easily be related to molecular structure, to derive the structure of the dimer, additional information is needed.

Chemical evidence comes from the reactions tested here: The pyrroline double bond cannot be involved in the dimerization reaction, because the biradical rate of formation and yield for MTSL is similar to that of MTSL‐P2, even though MTSL‐P2 lacks this double bond. Also, for MTSL and MTSL‐P2, the product must have a reducible cysteine bond, as the biradical EPR spectrum is abolished by TCEP. According to literature, TCEP is selective for disulfides and does not reduce the nitroxide group.^[^ ^28^ ^]^ Our experiments confirm this (see Experimental Section), proving that the disappearance of the biradical spectrum is not caused by partial nitroxide reduction in the biradical.

The proposed structure of the dimer (Figure 5) agrees with the base peaks from mass spectrometry. Consequently, the asymmetric dimer shown in Figure 5 fulfills all the criteria stated above and can be explained by the reaction pathway shown in Figure 5.

The reaction starts with the nucleophilic attack of a carbanion at the mesyl group of one MTSL molecule on the mesylated sulfur atom of a second MTSL molecule. The reaction proceeds with the mesyl‐group of the second MTSL as the leaving group, forming the hypothesized product. This nucleophilic substitution can be explained by the fact that the methanesulfoxide group is relatively acidic, while methanesulfinate is known to be an excellent leaving group. The reaction leads to a product that has the expected chemical properties and agrees with the sum formula from mass spectrometry of the reaction product.

Given that the reaction mechanism does not involve a radical step, the reaction is likely to occur also in other labeling reactions, thus also when fluorescent labels are attached to a cysteine‐bearing protein by a methanethiosulfoxide group, yet it was never reported. The reasons could be that fluorescent labels are more often linked by other groups, like the maleimide group, and that the labeling is often carried out at lower concentrations than spin‐labeling reactions. Also, fluorescence spectroscopy is not expected to give a clear dimer signature, unlike EPR spectroscopy in case of a biradical. Ultimately, any dimer product would be removed in the final labeling step when unreacted label is removed from the protein.

The question that remains is if the side reaction can be suppressed or avoided in spin‐labeling reactions. The disulfide linkage of the dimer makes this unlikely as breaking the disulfide bond of the dimer by a reductant would also decouple already coupled spin label from the protein, so it would not be useful. In this case, a switch to other linking groups^[^ ^3^ ^]^ or approaches, such as those described in refs. [7–9], could be the best option.

In conclusion, we show that the unwanted biradical reaction that hampers spin‐labeling reactions of proteins is an irreversible process, in which the unwanted product is formed in high yields. We propose a structure for this product that agrees with the mass obtained from mass spectrometry and the chemical properties observed in this study. So after four decades of mystery, we have elucidated the reaction that causes the formation of the biradical product in spin‐labeling reactions of proteins. We show that this biradical is a covalently bound dinitroxide, linked via a disulfide, or more precisely a sulfide‐sulfoxide bond.

## 4 Experimental Section

4.1

4.1.1

##### Description of Experiment

MTSL ((1‐oxyl‐2,2,5,5‐tetramethyl‐delta‐3‐pyrroline‐3‐methyl)methanethiosulfonate)and MTSL‐P2 ((1‐oxyl‐2,2,5,5‐tetramethylpyrrolidin‐3‐yl) methyl methanethiosulfonate) were purchased from Toronto Research Chemicals. Stock solutions were prepared in DMSO, MTSL concentration 24 mM, and stored at −20 °C. The samples were prepared by diluting the stock solutions in 10 mM TRIS‐HCl, buffer pH 7.4, for a final MTSL (resp. MTSL‐P2) concentration of 100 µM. Incubation was performed on a thermomixer (Eppendorf, Thermomixer comfort) at a temperature of 24 °C and shaking at 1000 rpm. A volume of 20 µL was extracted at selected time points for EPR measurement. TCEP (tris(2‐carboxyethyl)phosphine) was purchased from Sigma–Aldrich. To test reduction, a fivefold molar excess of TCEP was added to the incubating solution; the reaction was allowed to proceed for 30 min before EPR measurement. The EPR signal intensity (double integral) was measured before adding TCEP and after the end of the incubation time to be sure that no radical decay occurred after addition of TCEP and during the incubation time. For preparation of the ESI‐MS samples, the stock in DMSO was diluted to a final MTSL (resp. MTSL‐P2) concentration of 100 µM in ammonium acetate (NH_4_Ac) buffer, pH 7.4, and incubated in the same conditions (24 °C, 1000 rpm). Dimer formation and effect of TCEP were checked with EPR measurements before ESI‐MS analysis.

##### EPR Measurement Conditions

EPR spectra were acquired on an EMX EPR spectrometer (Bruker, Rheinstetten, Germany). Measurement conditions were as follows: microwave frequency: 9.88 GHz, modulation amplitude: 0.2 mT, modulation frequency: 100 kHz, power: 0.63 mW, time constant: 10.24 ms, conversion time: 10.24 ms. All measurements were performed at room temperature.

##### EPR Spectra Simulation

MATLAB (R2019a, The MathWorks, Inc., Natick, MA, USA) and the EasySpin package (5.2.35)^[^ ^29^ ^]^ were used for simulations of the EPR spectra. Spectra were simulated as the sum of monoradical and biradical nitroxides using the “pepper” function with isotropic g and hyperfine tensors, since biradical spectra can only be simulated by “pepper.” Parameters used for the simulation can be found in Table 1. The contribution (weight) of the monomer and biradical components to the simulation was adjusted to match the experimental spectral shape. Errors were determined by manually adjusting the contributions of the components until the simulation were visibly deviating from the experimental lineshape.

##### ESI‐MS

ESI‐MS samples were prepared as described in the “Description of Experiment” section above. Measurements were performed with an Q‐Exactive HF Orbitrap (Thermo Scientific) equipped with an electrospray ion source (ESI), injection of 2 µL of a 1 µM solution via Ultimate 3000 nano UPLC (Dionex) system, with an external calibration (Thermo Scientific). Source voltage of 3.5 kV, capillary temperature 275 °C, no sheath gas, resolution = 240.000 at *m*/*z* = 400. Mass range *m*/*z* = 160–2000 or until a maximum of 6000. Eluent used: MeCN:H_2_O (1:1 v/v) supplemented with 0.1% formic acid.

## Conflict of Interest

The authors declare no conflict of interest.

## Supporting information

Supplementary Material

## Data Availability

The data that support the findings of this study are available from the corresponding author upon reasonable request.
