# Supplementary material for: Identifying the Elusive Dimerization Product Interfering with Methylsulfonato‐Group Labeling of Cysteines in Proteins
Source: ChemistryOpen. 2025 Oct 7;15(1):e202500314. doi: 10.1002/open.202500314 (PMC12831924; doi:10.1002/open.202500314)
Supplement: Supplementary file 1 — Supplementary Material [file OPEN-15-e202500314-s001.pdf]

Supplementary information for

Identifying the elusive dimerization product interfering with methylsulfonato-group labelling of cysteines in proteins

Leonardo Passerini[a], René Dekkers[b], Karthick Babu Sai Sankar Gupta[b], Mark Overhand[b], Martina Huber*[a]

[a] L, Passerini, M. Huber
Department of Physics, Huygens-Kamerlingh Onnes laboratory
Leiden University
Niels Bohrweg 2, 2333 CA, Leiden, the Netherlands
Huber@physics.leidenuniv.nl

[b] R. Dekkers, K. B. S. S. Gupta, M. Overhand
Leiden insititute of chemistry
Leiden University
Einsteinweg 55, 2333 CC, Leiden, the Netherlands

submitted to Chemisty Open

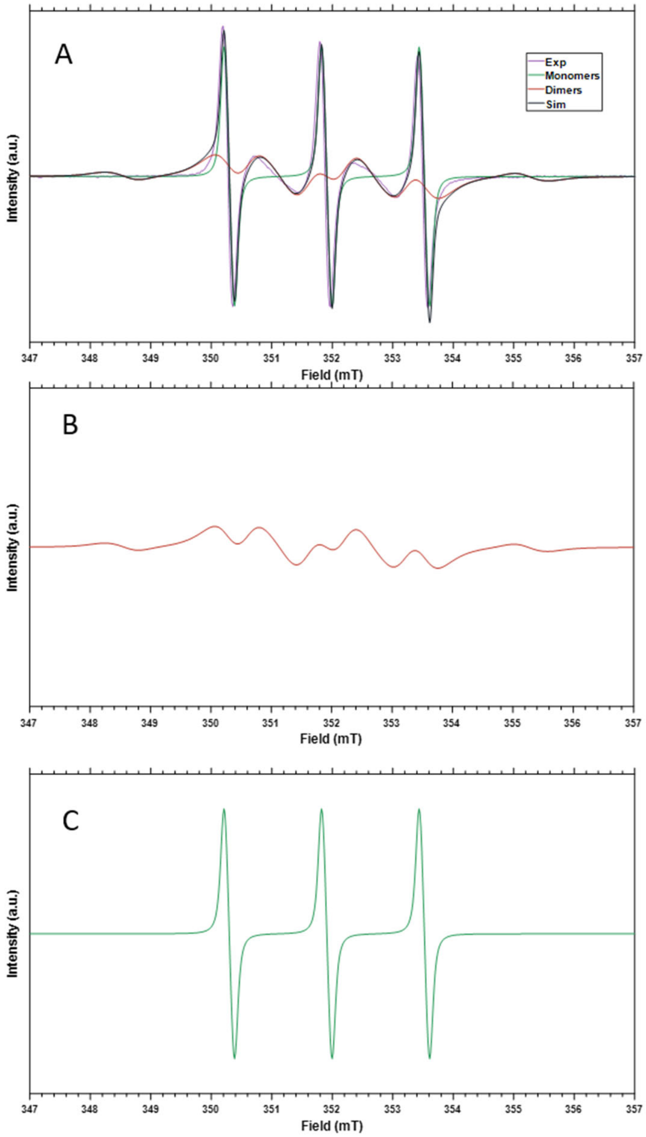

Figure SI.1 Formation of MTSL-P2 biradical monitored by EPR. EPR spectrum after incubation of MTSL-P2 in aqueous buffer. A) Spectrum, after nine hours. Purple : experimental spectrum, black : total simulation, orange : biradical component, green : mono radical component. B) Biradical component from A). C) Monoradical component. EPR spectra recorded as first derivative of absorption. The value of J used was selected solely to reproduce the spectrum between 350 and 354 mT. The simulation shows additional lines at 348 and 356 mT that are not experimentally measured, suggesting the J value used is not accurate.

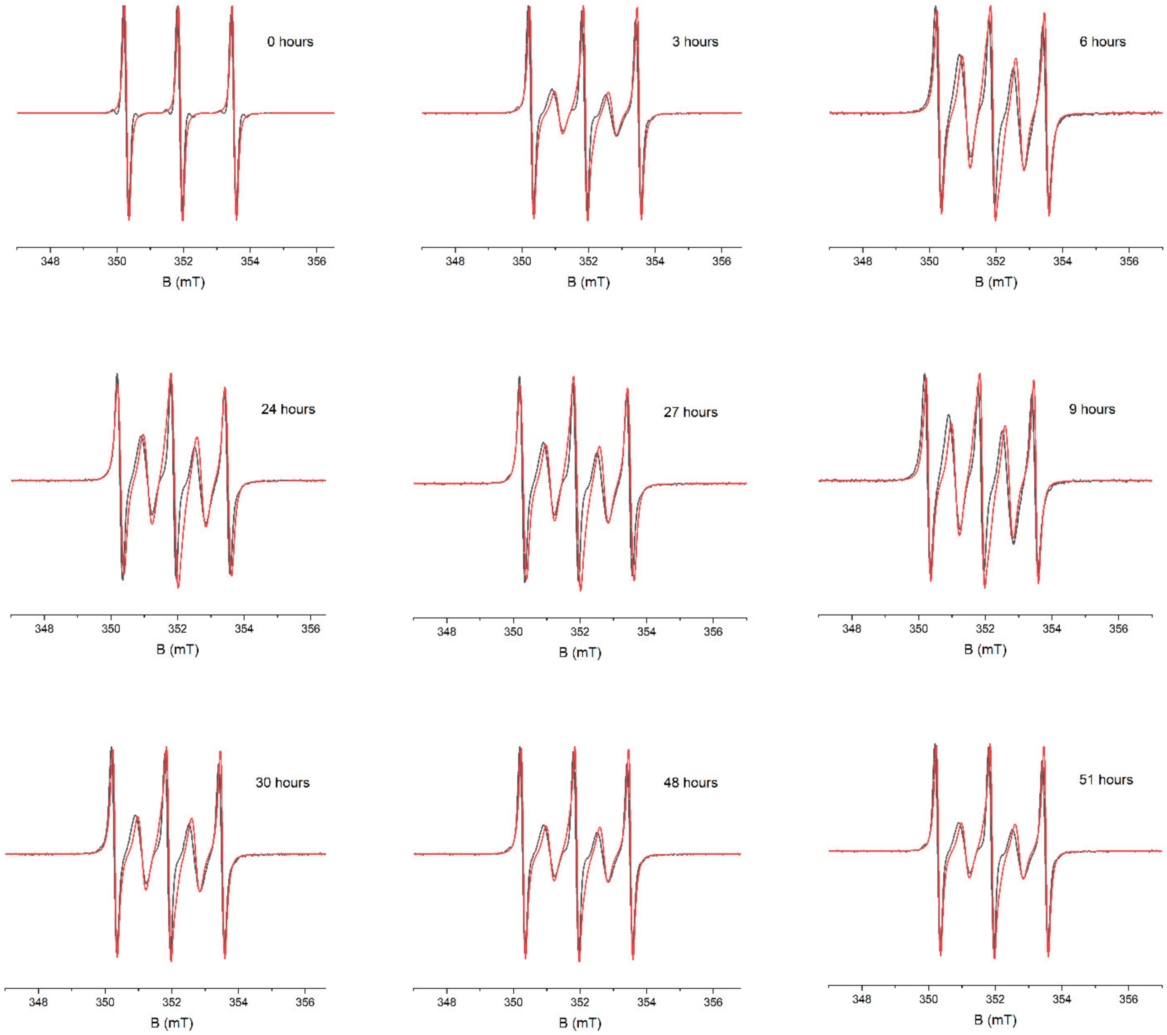

Figure SI.2 EPR spectra of MTSL acquired at different time points. Black: experimental spectrum. Red: simulation

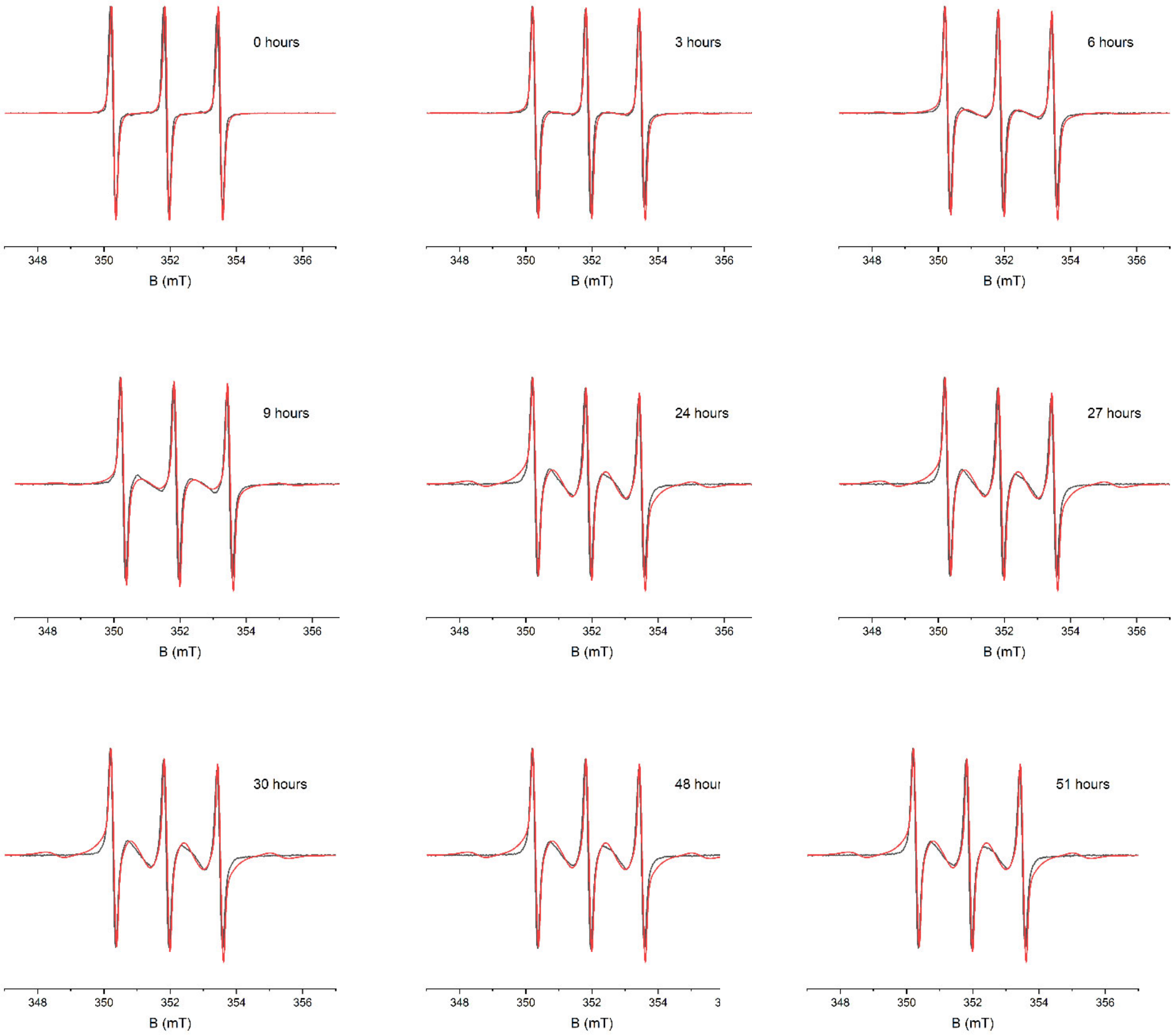

Figure SI.3 EPR spectra of MTSL-P2 acquired at different time points. Black: experimental spectrum. Red: simulation

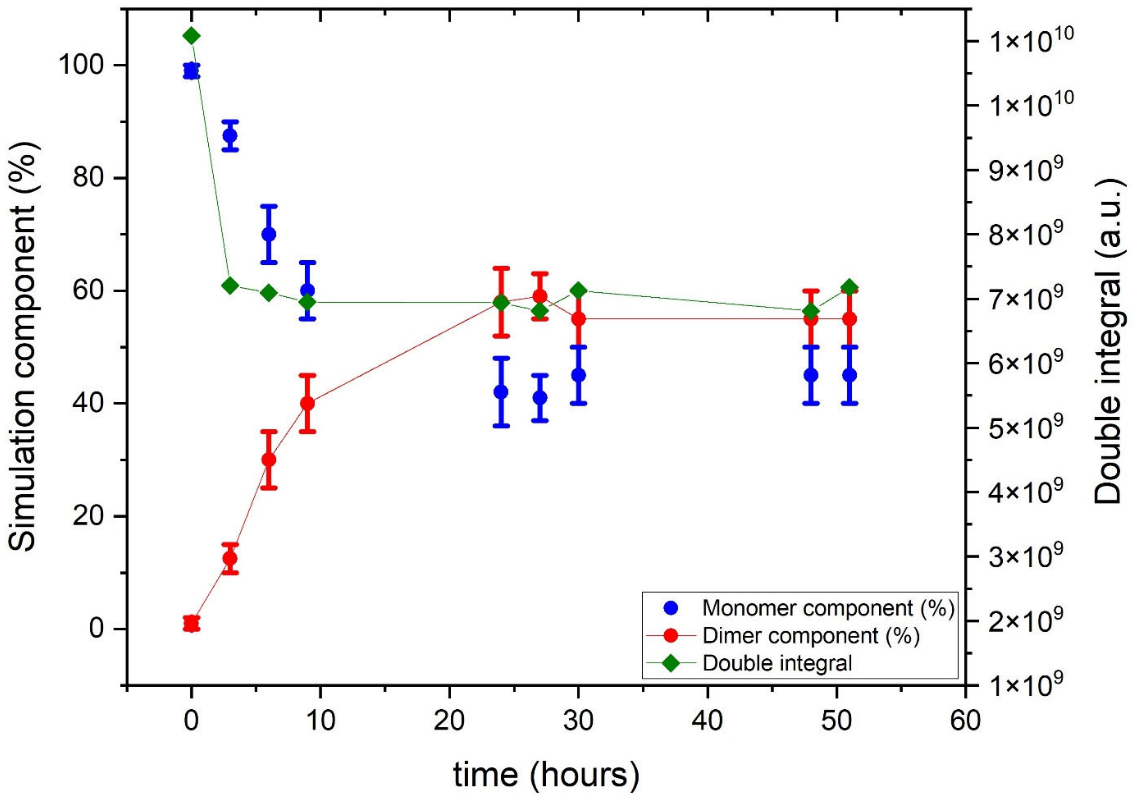

Figure SI.4. Development of MTSL-P2 biradical component over time. Contribution of the mono- and biradical component over the 51 hours of incubation. Blue : monoradical contribution, red : biradical contribution, green : total intensity of the EPR spectrum (double integral value). For details about error estimation, see materials and methods. Note that given that the simulation of the MTSL-P2 biradical spectrum gives additional lines that are not experimentally observed, the contribution measured through simulation of the biradical for MTSL-P2 is overestimated, and the monomer contribution is underestimated as a consequence.

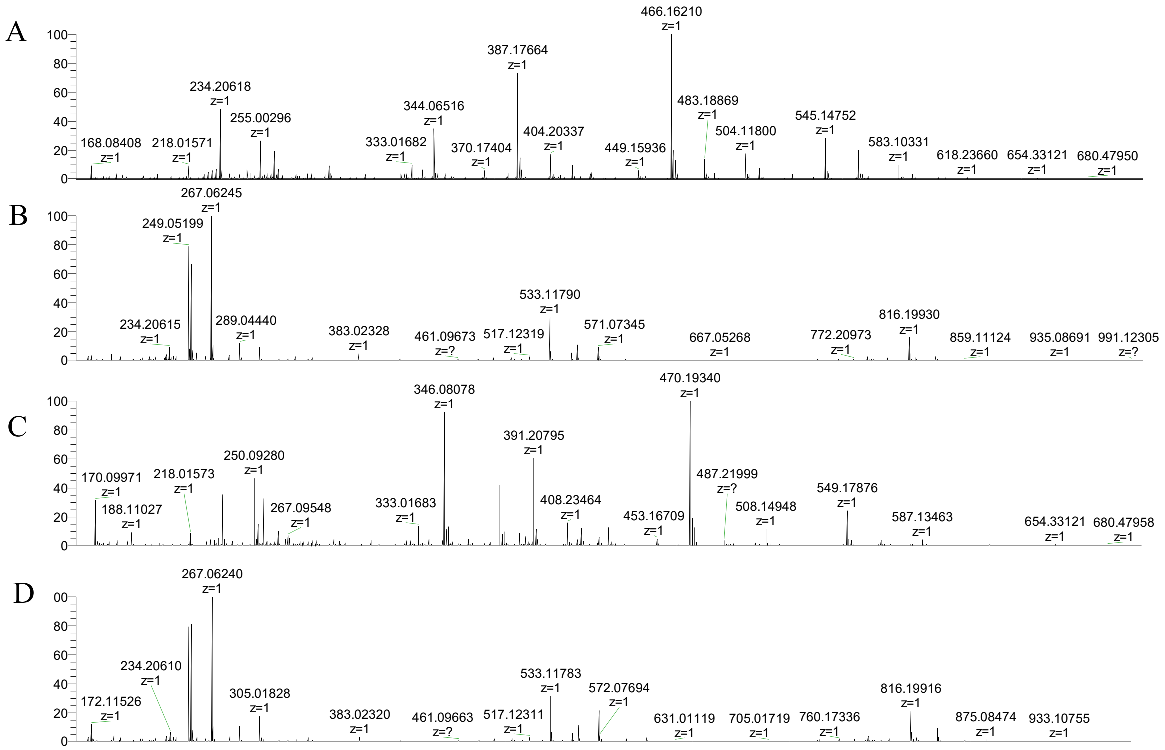

Fig. SI.5 MS-ESI spectra of MTSL and MTSL-P2. A): MTSL after three hours incubation. B): MTSL after three hours incubation and reaction with TCEP. C): MTSL-P2 after three hours incubation. D): MTSL-P2 after three hours incubation and reaction with TCEP.

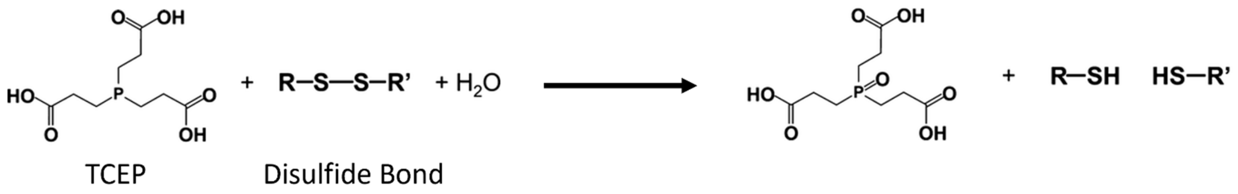

Fig. SI.6 Reaction scheme of the reduction of disulfide bonds by tris(2-carboxyethyl)phosphine) (TCEP).
